# A C++ expression system for partial differential equations enables generic simulations of biological hydrodynamics

**DOI:** 10.1140/epje/s10189-021-00121-x

**Published:** 2021-09-23

**Authors:** Abhinav Singh, Pietro Incardona, Ivo F. Sbalzarini

**Affiliations:** 1 Faculty of Computer Science, Technische Universität Dresden, Dresden, Germany; 2 Max Planck Institute of Molecular Cell Biology and Genetics, Dresden, Germany; 3 Center for Systems Biology Dresden, Dresden, Germany; 4 Cluster of Excellence Physics of Life, TU Dresden, Dresden, Germany

## Abstract

**Abstract:**

We present a user-friendly and intuitive C++ expression system to implement numerical simulations of continuum biological hydrodynamics. The expression system allows writing simulation programs in near-mathematical notation and makes codes more readable, more compact, and less error-prone. It also cleanly separates the implementation of the partial differential equation model from the implementation of the numerical methods used to discretize it. This allows changing either of them with minimal changes to the source code. The presented expression system is implemented in the high-performance computing platform OpenFPM, supporting simulations that transparently parallelize on multi-processor computer systems. We demonstrate that our expression system makes it easier to write scalable codes for simulating biological hydrodynamics in space and time. We showcase the present framework in numerical simulations of active polar fluids, as well as in classic simulations of fluid dynamics from the incompressible Navier–Stokes equations to Stokes flow in a ball. The presented expression system accelerates scalable simulations of spatio-temporal models that encode the physics and material properties of tissues in order to algorithmically study morphogenesis.

**Graphicabstract:**

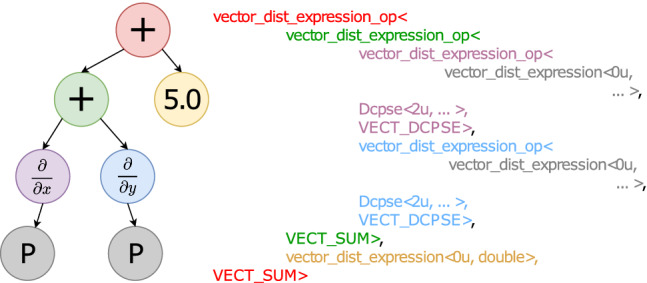

## Introduction

Numerical simulations of mathematical models of biological processes in space and time have become an integral part of studying the physical principles of living systems [1]. An important class of models on the scale of cells and multi-cellular tissues is continuum models, formulated as partial differential equations (PDE), that account for the mechanical properties of living matter [2–4]. Coupling biological hydrodynamics with biochemical regulation, such models have been successful at describing the physics underlying biological phenomena such as cell division [5], zygote polarization [6, 7], epithelial tissue folding [8], and cellular symmetry breaking [9].

Such mechano-chemical models of biological processes are usually nonlinear, either due to nonlinear chemical reaction terms or due to the hydrodynamics itself, e.g., the nonlinear polarity-velocity coupling in active polar fluids. While linear stability analysis provides important information about the phase space of these models [6, 9, 10], studying the full nonlinear dynamics requires numerical solutions or computer simulations of the models. Several simulation methods for active fluids have therefore been developed, including approaches based on finite-element methods [11, 12], hybrid particle-mesh methods [13], lattice-Boltzmann methods [14], and agent-based simulations [15]. The diversity and complexity of the corresponding computer simulation programs, however, highlights the need for a more intelligible syntax for large PDE models and for a cleaner separation between the numerical method and the PDE model in a software implementation.

Indeed, simulation software implementations are typically specific to a certain numerical method and a certain PDE model, with discretized differential operators hard-coded in explicit program statements. Changing the simulated model, for example to test new hypotheses, therefore usually requires rewriting much of the simulation program. This is not only time-consuming, but also creates challenges in terms of code structure and maintainability, as well as computational speed and parallel scalability. There is thus a need for generic simulation software platforms that separate the PDE model to be simulated from the numerical methods and that accelerate the implementation of efficiently scalable parallel computer programs.

Here, we present such a generic simulation environment based on the OpenFPM parallel computing framework [16]. It is based on a C++ template expression system for PDEs. This expression system allows specifying the PDE model to be simulated in near-mathematical notation and, importantly, independently of the specific numerical method to be used. The numerical method is transparently selected via includes and template parameters, which allows changing or further developing the numerical method without having to rewrite the model specification. It also allows changing the model, e.g., including additional terms in the PDE or changing the geometry of the solution domain, without having to change the implementation of the numerical solvers (albeit one may have to switch to a different solver). Our framework thus separates the specification of the PDE model and of the numerical discretization method. This leverages a principle from software engineering, *separation of concerns*, which is usually not exploited in numerical simulation codes.

The idea of achieving separation of concerns using template expressions is not new and has already been implemented decades ago, e.g., in the Par-EXPDE project [17]. The present system, however, is not tied to a certain computer architecture and is specifically designed for continuum simulations of biological hydrodynamics. It therefore supports both particle-based and mesh-based discretization, as well as hybrid particle-mesh methods [18]. Our template expressions transparently work for scalar, vector, and tensor fields, encapsulate time integration methods, and can be used to assemble equation systems for implicit solvers. Basing our system on OpenFPM renders it portable and ensures computational efficiency and scalability [16, 19].

After describing our framework, we illustrate how separation of concerns simplifies the implementation and maintenance of simulations of frequently occurring PDEs in biological hydrodynamics and renders simulation codes less error-prone. In the application examples, we show how our framework allows changing the simulated model by altering just a few lines of code. We do so by changing a simulation from solving the incompressible Navier–Stokes equations, to simulating active polar fluids in two dimensions, to Stokes flow in a three-dimensional ball. We also show how our framework allows to change the numerical method by providing examples using both grid-based finite-difference methods and mesh-free particle methods.

## The OpenFPM framework

The open framework for particles and meshes (OpenFPM) is a fully templated C++ library to implement scalable parallel simulation codes on CPUs and GPUs [16, 19]. It is available as open source from http://openfpm.mpi-cbg.de.

Codes written in OpenFPM have been shown to display computational performance and scalability on par with or exceeding those of state-of-the-art hand-written simulation programs [16]. But while hand-written codes often require years of development time, OpenFPM-based simulations can be instantiated within a few days to weeks. To achieve this, OpenFPM provides a higher level of abstraction. This hides the intricacies and specifics of a computer architecture (e.g., CPU vs. GPU) from the programmer by providing transparent data structures and operators [20–22].

**Fig. 1:**
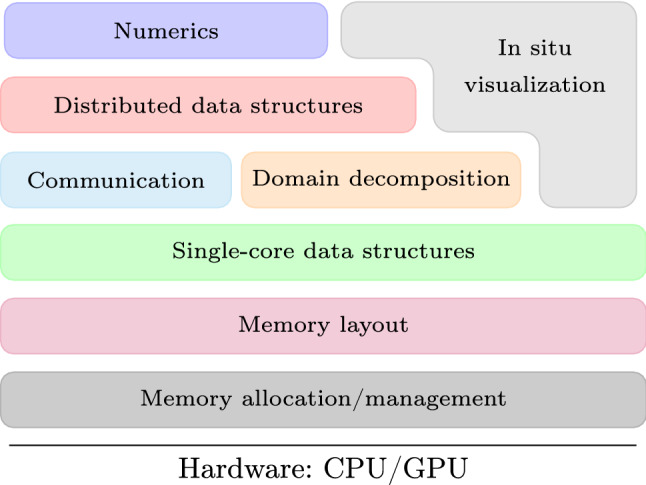
Layer structure of the OpenFPM software stack

These data and operator abstractions are internally distributed over the processors of a parallel computer. This distribution is invisible to the users, allowing them to focus on the simulated model and on the numerical method. Despite this high level of abstraction, OpenFPM maintains computational efficiency by leveraging C++ template meta-programming for compile-time generation of data-type, dimension-, and hardware-specific implementations of the abstractions. This enables abstractions over arbitrary data types, including user-defined C++ classes, and in arbitrary-dimensional spaces. Memory management, memory layout, and communication of data between nodes of a computer cluster, as well as to and from accelerators and the file system, are automatically handled by OpenFPM in an optimized way.

OpenFPM has a layered architecture as shown in Fig. 1, with each layer increasing the level of abstraction. This allows programmers to choose the level of abstraction appropriate to their needs by combining OpenFPM functions from different layers. The “lowest” (in the sense of closest to the operating system) layers provide abstractions for memory allocation and memory layout. This allows, e.g., to dynamically switch from an array-of-structures memory layout to a structure-of-arrays layout when transitioning data from the CPU to the GPU. Using these memory facilities, OpenFPM implements single-core data structures on the next-higher layer. These include vectors, tensors of arbitrary order, meshes, and sparse grids in arbitrary-dimensional spaces carrying arbitrary (also complex and composite as well as user-defined) data types. Using domain-decomposition and network-communication abstractions on the next layer, these data abstractions are composited to multi-core and distributed-memory versions that transparently scale across multiple computing devices, complete with the corresponding transparent iterators. Finally, the top layer implements a library of common numerical methods based on the OpenFPM data structures. Examples include finite-difference methods, multi-grid solvers, mesh-free kernel methods, preconditioners, and time- stepping methods for simulations. In addition, third-party libraries such as PETSc [23], Eigen [24], Odeint [25], and SPRNG [26] are wrapped and made available to the user under a uniform interface. A profiling interface and transparent in situ visualization of simulation results [27] complete the framework.

## C++ expression system for PDEs

The C++ programming language allows for custom expression systems to be built using template expression parsing techniques [28]. We design and implement such a system for expressing PDEs in near-mathematical notation as C++ code and to specify the numerical methods to be used for their discretization using include statements and encapsulated interfaces to numerical solvers.
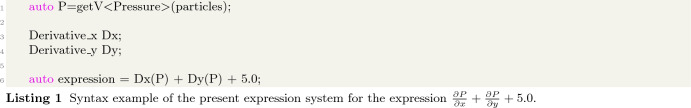


An example of the kind of code this allows one to write is shown in Listing 1. In line 1, the getV function creates an alias for the field Pressure stored on a set of discretization points called particles. This alias is then assigned to an object P with automatically inferred datatype. P can then be used as the pressure field discretized on particles. In lines 3 and 4, two derivative operators are declared, Dx and Dy of type Derivative_x and Derivative_y, respectively. Mathematical expressions can then be written as shown in line 6 for the example $\frac{\partial P}{\partial x} + \frac{\partial P}{\partial y} + 5.0$, providing a human-readable notation that automatically extends over the potentially many points in the set particles.

**Fig. 2:**
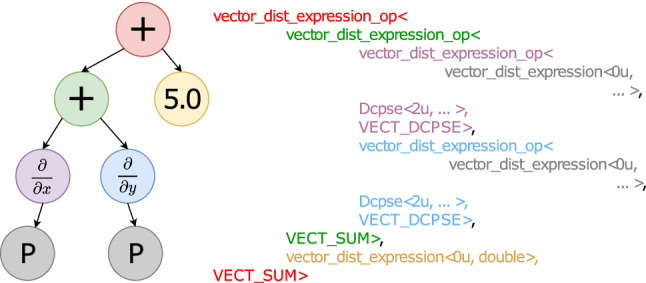
Tree representation of the expression $\frac{\partial P}{\partial x} + \frac{\partial P}{\partial y} + 5.0$ (left) and the resulting C++ template types (right)

These expressions are then analyzed and inlined by the compiler in order to generate the standard C++ code to evaluate them. For this, each expression is represented as a tree whose leaves (i.e., terminal nodes) are variables or numbers. Any interior (i.e., nonterminal) node of the tree represents one mathematical operator. As an example, the expression tree for line 6 in Listing 1 is shown in Fig. 2 on the left. This tree is subsequently translated to C++ class nodes by code production rules, which we adjust for our needs by operator overloading. To do so, we define templated class nodes representing the basic binary operators $+$, −, $*$, /, and derivative operators. These are inserted at the locations of the respective tree nodes in order to generate C++ types, as shown in Fig. 2 on the right.

Terminal nodes represent C++ objects linked to a particular continuous field in the PDE or to a numeric constant. They map to OpenFPM’s abstract data types, such as distributed vectors (vector_dist in Fig. 2) or meshes.

Each nonterminal node of the expression tree has 3 template parameters. For binary operators, the first template parameter is the expression on the left-hand side of the operator, the second parameter is the right-hand side expression of the operator, and the third parameter indicates the type of operation (e.g., $+$, −, $*$, /). An operator can either be applied to the result of another operator or to a field or constant in a terminal node. We implement binary operators and unary operators (e.g., derivatives). For the unary derivative operator, the second template parameter is the C++ class encapsulating the code used to discretize the derivative, i.e., the numerical method (Dcpse in Fig. 2).

For example, the production rule of the node $+$ in the expression $\frac{\partial P}{\partial x} + \frac{\partial P}{\partial y}$ is implemented by overloading the binary + operator for the two child nodes. In Fig. 2, both child nodes are of type vector_dist_expression_op$<\ldots>$ (purple and blue). This overloaded + operator returns the type for the sum of the two derivatives (green in Fig. 2). For objects of class Derivative, we additionally overload the () operator. This enables production rules for expressions like D(f) or D(f+g), denoting the derivative operator applied to the expression given inside the parenthesis as an argument to the unary () operator. This is how the purple and blue types in Fig. 2 are created, which in turn serve as input to the + operator.

In order to store the data of the simulation, each tree node defines a function value. Evaluating the function value of any node computes and returns the data at this node. This is done by recursively evaluating the operators on lower tree nodes as required by the natural tree traversal order. For computational efficiency, all nested calls to value functions along the tree are inlined. For the example from Listing 1, the data are the appropriately processed values of the field variable P at the locations of the discretization points in the set particles. Therefore, evaluating the function value of the root node of an expression tree produces the final result of the numerical computation of the given expression. For convenience, e.g., to simplify the construction of linear systems of equations, each node also provides a function value_nz. This function returns only the nonzero data of the respective node in a sparse matrix data structure.

Using this C++ template expression system, complex equations involving multiple discretized differential operators can be numerically evaluated in a transparent way. It is based on computational graphs constructed from C++ expression templates, which are then translated to distributed computations on OpenFPM data structures, such as OpenFPM vectors and grids, to generate scalable parallel simulations. Implicitly, these internally distributed C++ objects thus are symbolic references to data, which enables expression-based computing.
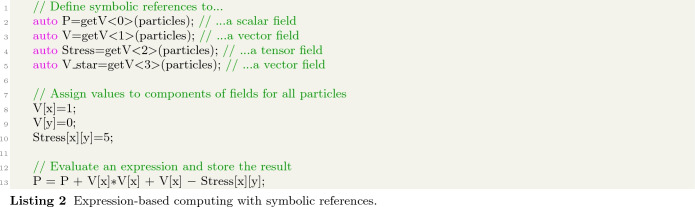


An example of expression-based computing is shown in Listing 2. It defines symbolic references for a scalar field (line 2), a vector field (line 3), and a tensor field (line 4) from the properties of corresponding datatype stored on a set of discretization points called particles. Scalar values are then assigned to the components of the vector field (lines 7, 8) and to a component of the tensor field (line 9) across all discretization points. Components are selected by their symbolic name passed to the overloaded unary [] operator. Finally, an expression is evaluated resulting in a scalar field stored in P (line 12). At compile time, this creates a computational graph and maps the computations as required for efficient run-time evaluation. This enables human-readable code for scalar, vector, and tensor expressions.

These expressions can also contain continuous derivatives, which are then automatically discretized using a numerical method as specified by an include statement. OpenFPM provides a library of frequently used numerical differentiation methods, including finite differences on regular Cartesian grids and the DC-PSE method [29] on irregularly scattered discretization points (see “Appendix A”). DC-PSE is a generalization of finite differences to arbitrarily distributed discretization points, which are then called *particles* as they do not need to form a lattice or mesh. This enables straightforward simulations in complex geometries [30] and Lagrangian simulations of hydrodynamics, where the particles move with the local flow velocity in order to simplify the governing equations [13]. Users can implement additional discretization methods as plug-ins, which can then be used in the present template expression system as well.

A code example involving derivatives is shown in Listing 3, where the DC-PSE operator library is imported in line 2. One can then instantiate correspondingly discretized versions of differential operators as shown in lines 6–10 for different examples. The first argument in the parentheses of the definition of a discretized derivative is the set of collocation points over which the operator is to be discretized. The second argument is a positive integer specifying the order of convergence for the discrete operator approximation. The third argument defines the cutoff radius for the operator support, i.e., the maximum distance around a discretization point where neighboring points contribute to the discrete operator. These discretized operators can then be used in symbolic expressions as detailed above in order to express derivatives (lines 13–15).
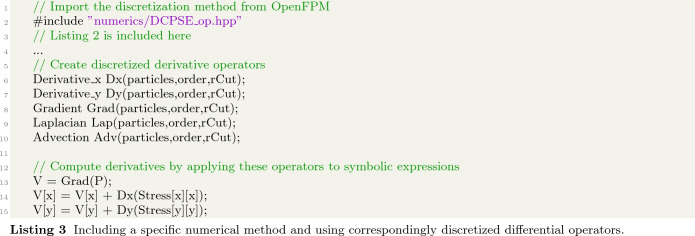


This defines an expression system that enables continuous models to be written in near-mathematical notation and to independently specify the numerical method that shall be used to discretize them. This cleanly separates the model definition from the implementation of the numerical methods, as was the main motivation for our work. However, it so far only allows to valuate explicit expressions, where the values can be computed successively along the expression tree.

For implicit equations, such direct evaluation is not possible. Instead, a numerical solver needs to be invoked to solve for the unknown variables in the linear or nonlinear equation system resulting from discretization. This is, e.g., the case when using implicit time-stepping methods or when solving for steady-state solutions. Both involve assembling the system matrix and the right-hand side vector of the linear system of equations to be solved. These are then passed to a numerical solver, such as a multi-grid solver, an LU decomposition, or a Krylov subspace solver. Many such solvers are implemented in numerical libraries like OpenFPM [16], PETSc [23], Eigen [24], and others, to which our framework provides access through a single, coherent interface.

An example of how to use implicit solvers is shown in Listing 4 to solve for the steady state of the Stokes equation. It imports a linear system solver based on DC-PSE in line 2. The solver is instantiated in line 9 for a simulation in two dimensions with two variables (2d2). The two equations for the two variables are defined in lines 18–21, complete with their right-hand sides. This uses the explicit symbolic expression system described above. These equations are then imposed for the two components x_comp and y_comp in the bulk of the simulation domain (lines 24 and 25. The boundary conditions are imposed in lines 27 and 28, here homogeneous zero-value Dirichlet boundaries. Finally, the numerical solver is executed in line 29, returning the solution V_star[x,y].
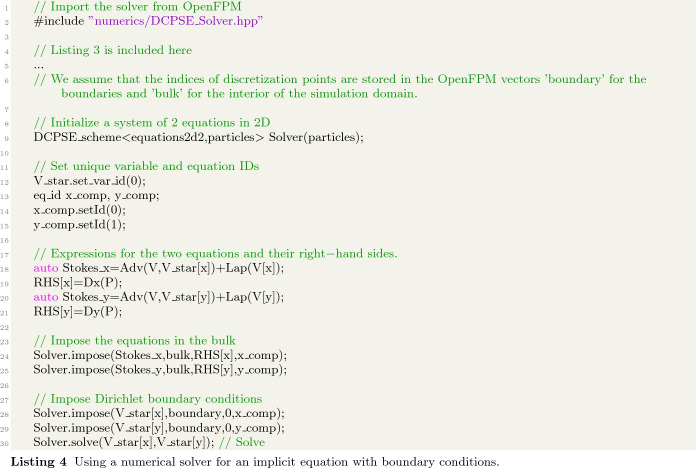


To change this from a DC-PSE solver for irregularly scattered discretization points to a finite-difference solver on a regular Cartesian grid, only the following lines change:
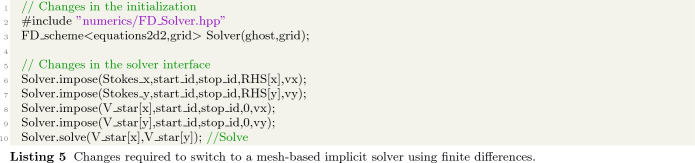


Instead of passing the OpenFPM distributed vectors bulk and boundary, containing the indices of the respective discretization points, we now pass an OpenFPM mesh grid, where start_id and stop_id are the grid point indices of the first and last points of the bounding boxes of the respective mesh regions. Alternatively, an OpenFPM mesh iterator can be used. All symbolic expressions for the PDEs to be solved remain unchanged, despite the fact that we now use a fundamentally different numerical method, namely a Cartesian mesh finite-difference solver instead of a mesh-free DC-PSE solver

In summary, we provide a C++ template expression system for numerically solving PDEs using both explicit and implicit methods. Our expression systems cleanly separates the definition of the model to be simulated from the implementation of the numerical methods used to do so. This allows implementing new solvers or changing the model without having to touch the respective other parts of the code. It also provides an almost mathematical notation for PDEs in C++ simulation program codes. For implicit equations, numerical solvers from different libraries are encapsulated under a common interface. The code generated by our expression system uses native distributed data structures from OpenFPM. This enables the resulting simulations to run in parallel on shared- and distributed-memory computers as well as on graphics cards. Users also benefit from the advanced visualization capabilities of OpenFPM, e.g., to store simulation results in portable VTK files or to remotely (over network) view the progress of a running simulation using *in situ* visualization [27].

## Application examples

We test our implementation and demonstrate the use of the presented expression system on three benchmark problems: First, we consider the incompressible Navier–Stokes equations, describing fluid flow at length scales where inertial forces play a role. Examples include air flow in the lungs [31] and blood flow in the heart [32, 33]. As a test case, we consider the nonlinear lid-driven cavity problem, which is a classic benchmark to check a simulation code’s ability to solve for steady-state incompressible flow. As an algorithmic novelty, we use our expression system to implement a mesh-free simulation with pressure correction. We validate this novel solver by comparing against data from Ghia et al. [34]. Further, we use this test case to evaluate how many lines of code need to be edited in order to change the mesh-free simulation to a finite-difference simulation on a regular Cartesian grid.

Second, we adapt the mesh-free solver with incompressible pressure correction to simulating the time-resolved dynamics of active polar fluids in two dimensions. These coupled PDEs describe the mechanics of active biological materials, such as the actomyosin cortex in cells [5, 6, 10], in the long-time hydrodynamic limit. We use this test case to demonstrate how to use our expression system to discretize PDEs in a Lagrangian frame of reference. We validate the resulting simulation code in a convergence study showing the correct scaling of the numerical error. We further use this second test case to benchmark the scalability of the resulting OpenFPM simulation on a multi-core computer, illustrating how well the code runs when distributed over multiple CPU cores.

As a third application example, we consider Stokes flow in a three-dimensional ball to demonstrate the versatility of the expression system for PDEs in non-Cartesian domains without rewriting the PDEs themselves. We validate this case by showing convergence to the analytical solution.

### Incompressible Navier–Stokes

As a first test case we consider the nonlinear lid-driven cavity problem governed by the incompressible Navier–Stokes equations in the unit square $[0,1]^2$ with the top boundary (i.e., the “lid”) moving at constant velocity ${\mathbf {v}}_b = (1,0)^\top$ and the rest of the boundaries having no-slip boundary conditions. The governing equations are:

1a $$\begin{aligned}&{\mathbf {v}}\cdot (\nabla {\mathbf {v}})-\frac{1}{\text {Re}}\mathrm {\Delta } {\mathbf {v}}=-\nabla \Pi \end{aligned}$$

1b $$\begin{aligned}&\nabla \cdot {\mathbf {v}}=0 \end{aligned}$$

1c $$\begin{aligned}&{\mathbf {v}}(x_b,y_b)=(0,0), \text { except } {\mathbf {v}}(x_b,1)=(1,0)\, , \end{aligned}$$

where $x_b$, $y_b$ are the coordinates of the boundary, $\Pi$ is the pressure, and $\text {Re}$ is the Reynolds number. We numerically solve these equations in primitive variables, velocity, and pressure. The incompressibility condition in Eq. (1b) is imposed using a pressure-correction scheme [35]. We discretize the differential operators in space using DC-PSE [29], which has previously been used to solve Navier–Stokes problems using velocity–vorticity correction [36, 37]. However, to our knowledge, it has never been used in a pressure-correction algorithm.

**Fig. 3:**
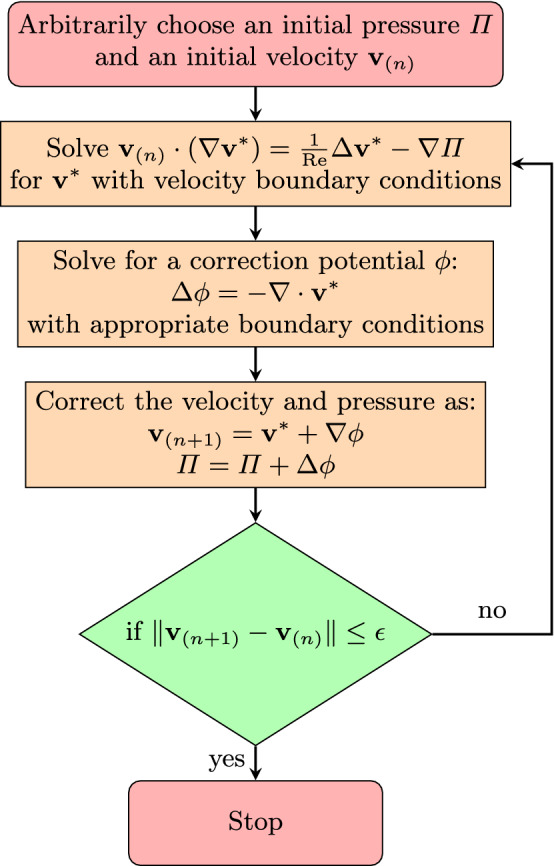
Flow diagram of the pressure-correction algorithm for steady-state incompressible Navier–Stokes simulations with user-provided numerical tolerance $\epsilon$

**Fig. 4:**
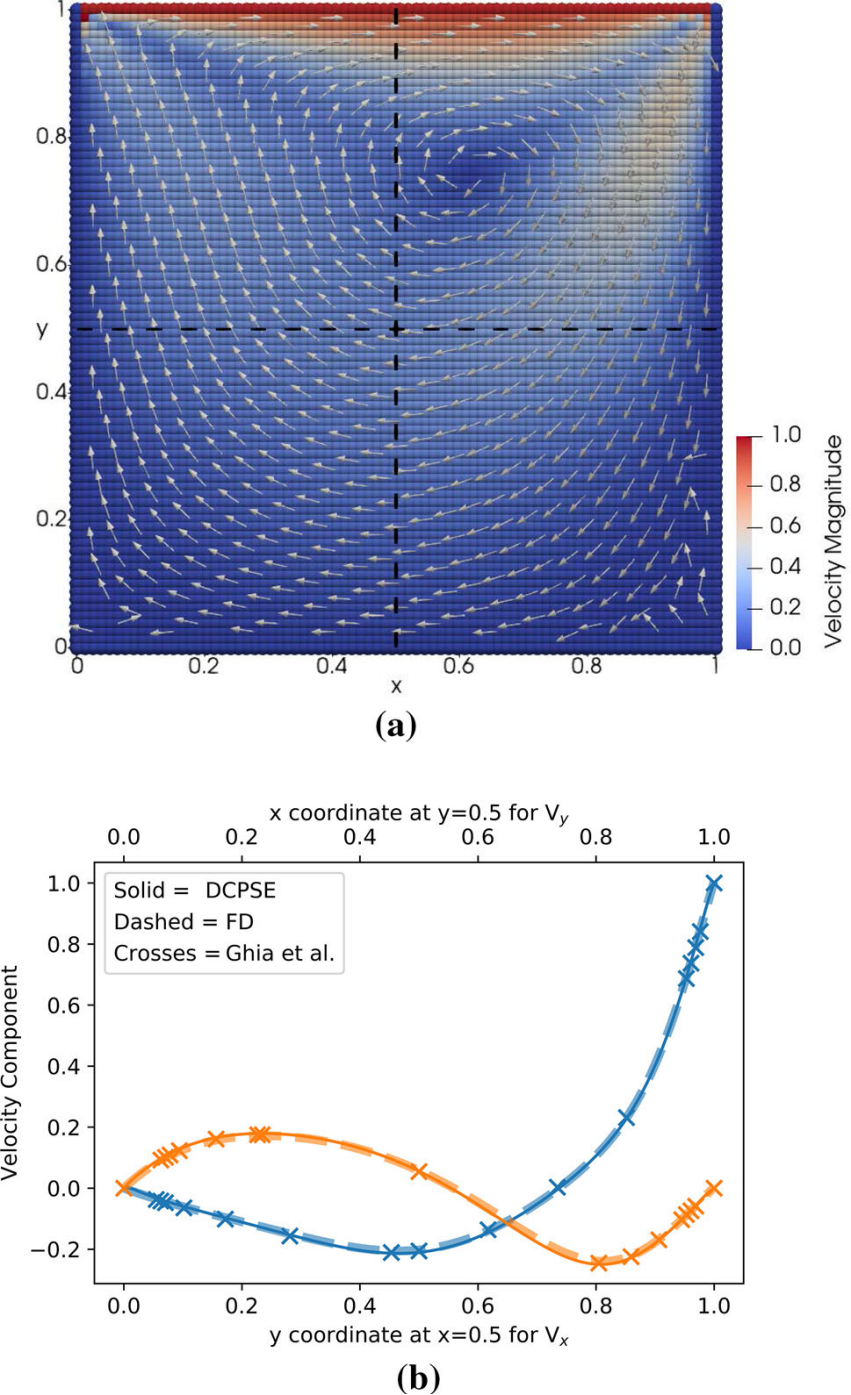
Nonlinear lid-driven cavity problem of Reynolds number $\text {Re}=100$ solved on an $81\times 81$ grid using the pressure-correction algorithm from Fig. 3. **a** Visualization of the velocity magnitude (color) and direction (arrows) as computed by the implicit DC-PSE solver in the two-dimensional simulation domain. **b** Velocity *x*-component (blue) along the vertical line $x=0.5$ (dashed in **a**) and *y*-component (orange) along the horizontal line $y=0.5$. We compare the present solution computed using second-order DC-PSE (solid lines) or finite differences (FD, dashed lines) with the available reference data from Ghia et al. [34] (crosses) for the same Reynolds number

Pressure correction [35] is a method from computational fluid dynamics to impose the incompressibility condition in numerical solutions of the incompressible Navier–Stokes equation. Due to the nonlinearity of Eq. (1a) in ${\mathbf {v}}$, numerical solutions generally violate incompressible continuity Eq. (1b), i.e., the flow velocity field is not guaranteed to be divergence-free. The idea of pressure correction is to use the pressure field as a Lagrange multiplier, which is determined such that the velocity becomes exactly divergence-free. This algorithm is illustrated in Fig. 3 for steady-state solutions. It iteratively solves for the velocity field with a given pressure field, then computes the correction potential for incompressibility, corrects the velocity and the pressure accordingly, and iterates until convergence. This simulation algorithm has previously been used in conjunction with moving least squares discretization methods [38].

We discretize the square two-dimensional domain with collocation points arranged on a regular Cartesian lattice of $81\times 81$ points. We use DC-PSE operators of convergence order 2 with an interaction cutoff of 3.1 grid cells to build the system matrix. We use the KSPGMRES solver from PETSc to solve the system for the steady-state solution. When implemented in our C++ expression system, the complete algorithm requires 156 lines of code, not counting comments and empty lines. A visualization of the simulation result is shown in Fig. 4a. To validate the simulation, we compare with reference simulation data from Ghia et al. [34], which is available for Reynolds number $\text {Re=100}$ for the *x*-component of the velocity along a vertical line across the domain at $x=0.5$ and the *y*-component of the velocity along a horizontal line across the domain at $y=0.5$. Our results in Fig. 4b are indistinguishable from the reference solution.

The lid-driven cavity is a popular test case because it is challenging for numerical methods and yet the laminar solution is steady. The main numerical challenges are the nonlinearity of the equation and the fact that the domain has sharp corners where stagnation points or recirculation vortices develop. Since only the top lid moves, with lateral and bottom walls stationary, a global vortex develops in the flow field whose center location depends on the Reynolds number. Indeed, when we repeat our simulation for the Stokes limit $\text {Re}=0$, the center of the vortex is on the vertical middle axis at $x=0.5$, while for $\text {Re}=100$ it is shifted towards the top-right quadrant (Fig. 4a).

We further use this test case to quantify how difficult it is to change the simulation to using a different numerical method. Therefore, we edit the code to use finite-difference stencils instead of DC-PSE. This requires deleting the boundary particle detection, changing the declaration of the differential operators, and importing the finite-difference OpenFPM library. Altogether, it requires changing 21 lines of code. The overall simulation logic and all PDE expressions remain unchanged, and the solution still matches the benchmark data (Fig. 4b, dashed lines). This illustrates how the present expression system can accelerate the testing of alternative numerical methods in a simulation program.

### Viscous active polar fluids

In the second test case we implement a solver for the viscous active polar fluid equations [4] in two dimensions and compare with a benchmark simulation [13]. The nonlinear, nonequilibrium hydrodynamics of viscous active polar fluids is described by the following set of PDEs in Einstein summation notation:

2a $$\begin{aligned}&\frac{\mathrm {D} p_{\alpha }}{\mathrm {D} t}=\frac{h_{\alpha }}{\gamma }- \nu u_{\alpha \beta } p_{\beta }+\lambda \varDelta \mu p_\alpha + \omega _{\alpha \beta } p_{\beta } \end{aligned}$$

2b $$\begin{aligned}&\partial _{\beta } \sigma _{\alpha \beta }-\partial _{\alpha } \Pi =0 \end{aligned}$$

2c $$\begin{aligned}&\partial _{\gamma } v_{\gamma }=0\end{aligned}$$

2d $$\begin{aligned}&2 \eta u_{\alpha \beta } =\sigma _{\alpha \beta }^{(s)}+\zeta \varDelta \mu \left( p_{\alpha } p_{\beta }-\frac{1}{2} p_{\gamma } p_{\gamma } \delta _{\alpha \beta }\right) \nonumber \\&\qquad \qquad -\frac{\nu }{2}\left( p_{\alpha } h_{\beta }+p_{\beta } h_{\alpha }-p_{\gamma } h_{\gamma } \delta _{\alpha \beta }\right) \end{aligned}$$

for the spatial components $\alpha ,\beta ,\gamma \in \{x,y\}$ denoted by subscripts. Equation (2a) governs the dynamics of the polarity field ${\mathbf {p}}=(p_\mathrm {x},p_\mathrm {y})^\top$. The Lagrangian (or material) derivative is defined as usual:

3 $$\begin{aligned} \frac{\mathrm {D} p_{\alpha }}{\mathrm {D} t}=\frac{\partial p_{\alpha }}{\partial t}+v_{\gamma } \partial _{\gamma } p_{\alpha }. \end{aligned}$$

The constant $\gamma$ is the rotational viscosity, $\nu$ is the coupling coefficient for polarity and mechanical stress, and $\lambda$ is the coefficient coupling the active chemical potential $\varDelta \mu$ with the polarization dynamics; $u_{\alpha \beta }=\frac{1}{2}\left( \partial _{\alpha } v_{\beta }+\partial _{\beta } v_{\alpha }\right)$ is the strain rate tensor and $\omega _{\alpha \beta }=\frac{1}{2}\left( \partial _{\beta } v_{\alpha }-\partial _{\alpha } v_{\beta }\right)$ the vorticity tensor. The so-called molecular field $h_\alpha$ is the variational derivative of the free energy density

4 $$\begin{aligned} f=\frac{K_\mathrm {s}}{2} (\nabla \cdot {\mathbf {p}})^2 + \frac{K_\mathrm {b}}{2} (\nabla \times {\mathbf {p}})^2 + {\frac{h^0_{\Vert }}{2}\Vert {\mathbf {p}}\Vert ^2} \end{aligned}$$

with elastic constants $K_\mathrm {s}$ and $K_\mathrm {b}$ for the splay and bending coefficients, respectively. The Lagrange multiplier $h^0_{\Vert }$ enforces unit magnitude of the polarity. Equation (2b) is the force balance with the total stress tensor $\sigma _{\alpha \beta }=\sigma ^{(\mathrm {p})}_{\alpha \beta }+\sigma ^{(\mathrm {a})}_{\alpha \beta }$ as the sum of passive ($\mathrm {p}$) and active ($\mathrm {a}$) stresses (see “Appendix B” for details) and the pressure $\Pi$. Equation (2c) is the incompressibility condition on the velocity field ${\mathbf {v}}$. Equation (2d) is the constitutive stress–strain relation with the symmetric stress $\sigma ^{(\mathrm {s})}_{\alpha \beta }$ (see “Appendix B”), the viscosity $\eta$, the coefficient $\zeta$ coupling material stress to mechano-chemical activity, and the Kronecker delta $\delta _{\alpha \beta }$.

**Fig. 5:**
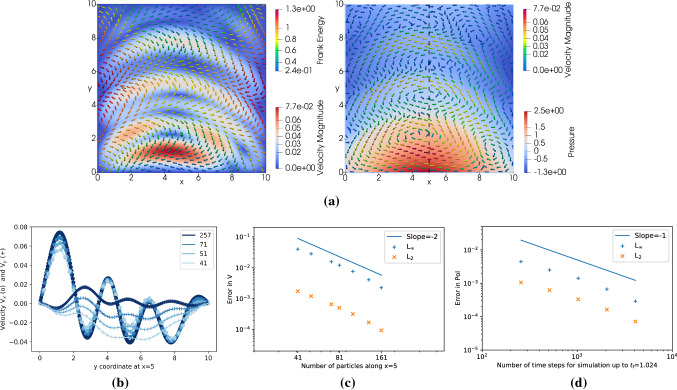
Visualization and grid convergence of the velocity and polarity fields for the two-dimensional viscous active polar fluid simulation. **a** Left: visualization of the polarity field (arrows colored by Frank free energy) and of magnitude of the velocity (background color). Right: visualization of the velocity field (arrows colored by magnitude) and of the pressure (background color). Both panels are at time $t=2\cdot 10^{-6}$. **b** Profile of the *x*- () and *y*-components (+) of the velocity along the black dashed line in **a** at $x=5$ for initial grids of increasing resolution from $41\times 41$ to $257\times 257$ (grayscale, see inset legend) showing convergence of the solution. **c** Spatial grid convergence of the error in the velocity vectors compared to a highly resolved simulation of $257\times 257$ particles. Both the $L_2$ and $L_\infty$ norms of the absolute error at $t=2\cdot 10^{-6}$ over all particles are shown. **d** Temporal grid convergence of the error in the polarity vectors for an increasing number of time steps to reach time $t=1.024$, compared against a highly resolved simulation with $16\,384$ time steps on $41\times 41$ particles. The solid lines in **c** and **d** show the theoretically expected error scaling

This formulation of an active polar material model is valid in the hydrodynamic limit, where the material behaves as a viscous fluid, and elastic stresses are considered to be relaxed [39]. Already these equations (see “Appendix B” for component-wise notation) are sufficiently complex to demonstrate the use of the present template expression system. They describe a nonequilibrium, nonlinear incompressible Stokes flow problem with 4 unknown fields (the scalar field $\Pi$, the vector fields ${\mathbf {v}}$ and ${\mathbf {p}}$, and the tensor field $\varvec{\sigma }$) and 9 terms on the right-hand side altogether. For PDEs of such complexity, our expression system is particularly useful, as it makes the simulation code more readable and its implementation less error-prone.

We discretize all fields on Lagrangian particles that move with the velocity ${\mathbf {v}}$ of the flow. Differential operators are consistently approximated over the irregularly distributed set of moving collocation points using DC-PSE [29]. This enables an important difference to previous approaches [13], which is that we do not need to interpolate the Lagrangian particles to a regular grid at every time step. Instead, we solve the implicit equation for the velocity directly on the irregularly distributed particles, using DC-PSE operators to build the system matrix and the KSPGMRES linear system solver from PETSc [23] to solve the system.

We also extend the simulation to solve for the time dynamics of the fields using an explicit Runge–Kutta time stepping method of order 4 for the polarity field and explicit Euler time stepping for moving the particles, both with time step size $\delta t = 2\times 10^{-7}$. Using the present expression system, the entire simulation code is 340 lines long, reusing 50 lines from the lid-driven cavity solver. By changing 20 lines of the code, the numerical method can be changed from a DC-PSE discretization on moving Lagrangian particles to a finite-difference simulation on a static Cartesian grid.

We verify our implementation for the benchmark problem from Ref. [13], where a staggered-grid finite-difference scheme has been used in conjunction with Lagrangian particles and remeshing. We therefore solve Eqs. (2) in the square domain $[0,10]^2$ with edge lengths $L_x=L_y=10$ with initial condition for the polarity

5 $$\begin{aligned} {\mathbf {p}}(x,y,0)=\left( \begin{matrix} \sin \big (2\pi (\cos \left( \frac{2 x - L_x}{ L_x}\right) -\sin \left( \frac{2 y - L_y}{ L_y}\right) \big ) \\ \cos \big (2\pi (\cos \left( \frac{2 x - L_x}{ L_x}\right) -\sin \left( \frac{2 y - L_y}{ L_y}\right) \big ) \end{matrix}\right) , \end{aligned}$$

boundary conditions for polarity and velocity

6 $$\begin{aligned} {\mathbf {p}}(x_b,y_b,t)&={\mathbf {p}}(x_b,y_b,0), \end{aligned}$$

7 $$\begin{aligned} {\mathbf {v}}(x_b,y_b,t)&=(0,0)^\top , \end{aligned}$$

and parameters $\eta =1$, $\nu =-0.5$, $\gamma =0.1$, $\zeta =0.07$, $\lambda =0.1$, $K_{\mathrm {s}}=1$, $K_{\mathrm {b}}=1$, $\varDelta \mu =-1$. This problem has been previously used as a test case to confirm consistency of numerical methods for active and nematic fluids [13]. It models a thin active polar viscous fluid in a square dish, such as an *in vitro* actomyosin film, with no-slip velocity boundary conditions and the filaments fixed (i.e., anchored) at the boundaries.

The simulation proceeds by solving for the steady-state velocity using the given polarity at the initial time. The collocation points (particles) are initially placed on a regular Cartesian grid. From there, the particles are advected by the flow velocity ${\mathbf {v}}$, and the polarity field ${\mathbf {p}}$ is evolved according to the Lagrangian derivative in Eq. (2a). Since the particles move, the DC-PSE operators are recomputed at each time step, and all steps are repeated until the final time. For this simulation, we expect first-order convergence in time, limited by the Euler method used to move the particles, and second-order convergence in space given by the order of the DC-PSE operators.

The results are shown in Fig. 5. Figure 5a visualizes the simulated polarity, velocity, and pressure fields at time $t=2\cdot 10^{-6}$, which can directly be compared with Fig. 4d,g in Ref. [13]. The initial harmonic map of the polarity creates a strong gradient in the Frank free energy, which is unfavorable due to the oscillation (high bending energy) and the fixed-polarity boundary condition. Consequently, the system is mainly driven by the equilibrium stresses, and the force balance results in the flow field visualized on the right with a nonmonotonic pressure. Since the active chemical potential $\Delta \mu$ is assumed to be constant, it does not significantly affect the behavior of the active fluid. However, increasing the activity leads to a faster flow with slower relaxation of the polarity field due to the free energy. Changing the sign of the activity reverses the flow direction, and the fluid starts behaving as an extensile material.

The initial and boundary conditions used here render this test case numerically challenging, because the oscillations in the polarity field cause multiple small vortices in the velocity field (see Fig. 5a, right). We validate the consistency of our simulation by showing grid convergence of the velocity field along the vertical center axis in Fig. 5a. The two components of the velocity along this line are plotted in Fig. 5b for initial grid resolutions increasing from $41\times 41$ to $257\times 257$ particles. While the *x*-component (circles) of the velocity converges rapidly, the *y*-component (pluses) is more sensitive to the numerical resolution. However, both components converge with the expected convergence rate of 2, as shown for the full error norms of the velocity vector field in Fig. 5c. Finally, we also validate grid convergence in time for the polarity field relaxing until final time $t=1.024$. The results in Fig. 5d show the expected first-order convergence in time, due to the Euler time-integration method used.

**Fig. 6:**
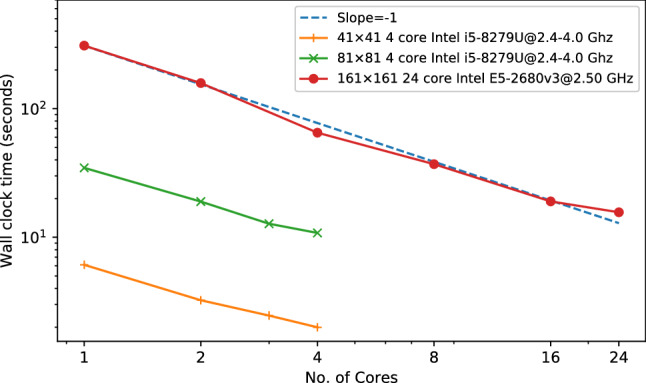
Strong scaling of the active polar fluid simulation for different initial grid resolutions. We plot the wall-clock time taken to complete 10 simulation time steps on an increasing number of CPU cores for two different processor models (inset legend). The ideal scaling is indicated by the dashed blue line

We also use this test case to demonstrate that the OpenFPM code generated by our template expression system is parallel and scales well on multi-core computers. For this, we perform a strong scaling experiment of the present active polar fluid simulation for initial grids of different sizes, but without rewriting or manually tuning any of the code. The result in Fig. 6 shows a parallel efficiency of 87% when scaling the code up to all 24 cores of an Intel Xeon E5-2680v3 processor at 2.5 GHz clock frequency. Further, on a 4-core Intel i5 mobile consumer CPU, we measure a parallel efficiency of 70% (strong scaling) even when clock boost is enabled for single-core tasks.

**Fig. 7:**
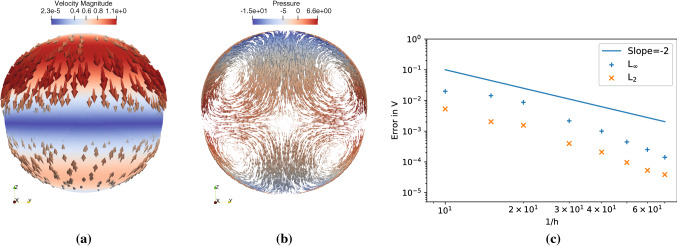
Visualization and convergence for Stokes flow inside a three-dimensional unit ball. **a** Visualization of the flow velocity (arrows colored by magnitude) in comparison with the analytical solution for mode $l=2$ (magnitude: solid background color). **b** Velocity (arrows) and pressure (color) visualized in the $y-z$ plane cut through the ball’s center. **c** Convergence plot showing the $L_2$ and $L_\infty$ norms of the absolute error in the velocity field computed against the analytical solution for $l=2$ for different average inter-particle spacing *h*. The solid line shows the theoretically expected scaling

### Stokes flow in a three-dimensional ball

In the third test case we consider a simulation in a non-Cartesian geometry, where we validate against an analytical solution. For this, we numerically solve the incompressible steady-state Stokes flow equations

8a $$\begin{aligned} \mathrm {\Delta } {\mathbf {v}}= & {} \nabla \Pi \, , \qquad {\mathbf {v}}\in \Omega \backslash \partial \Omega \end{aligned}$$

8b $$\begin{aligned} \nabla \cdot {\mathbf {v}}= & {} 0 \end{aligned}$$

in the closed unit ball $\Omega =\overline{B_1}(0)\subset {\mathbb {R}}^3$ centered at the origin. We solve these equations for the velocity ${\mathbf {v}}$ and the pressure $\Pi$ by imposing velocity boundary conditions on the surface of the ball, given by the vector spherical harmonics with unit amplitude for the mode $l=2$, $m=0$. The analytical solution for this problem is then known [40] as:

9a $$\begin{aligned} {\mathbf {v}}&=\sum \limits _{l=0}^{\infty }\sum \limits _{m=-l}^{l} u_{l m}^{r}(r) {\mathbf {Y}}^{(l m)}+u_{l m}^{(1)}(r) \mathbf {\Psi }^{(l m)}\nonumber \\&\quad +u_{l m}^{(2)}(r) \mathbf {\Phi }^{(l m)} \end{aligned}$$

9b $$\begin{aligned} \Pi&=\sum _{l=0}^{\infty } \sum _{m=-l}^{l} p_{l m}(r) Y_{l m}, \end{aligned}$$

where ${\mathbf {Y}}^{(l m)},$ $\mathbf {\Psi }^{(l m)}$, and $\mathbf {\Phi }^{(l m)}$ are the vector spherical harmonics, $Y_{l m}$ is the scalar spherical harmonic, and $u_{l m}^{r}(r)$, $u_{l m}^{(1)}(r)$, $u_{l m}^{(2)}(r)$, and $p_{l m}(r)$ are coefficients determined from the velocity boundary condition.

We use DC-PSE to discretize the differential operators in space and to build the system matrix. We then use the KSPGMRES solver from PETSc [23] as encapsulated by our expression system to numerically solve the resulting linear system of equations with pressure correction (see Section 4.1) to impose the incompressibility condition. This validates the mesh-free pressure correction scheme using DC-PSE also in a simulation domain with curved boundary.

Implementing this simulation using the present C++ expression system, the OpenFPM code is 180 lines long, which includes the initialization of the boundary conditions from numerically computed vector spherical harmonics. We validate the simulation by comparing against the analytical solution for different modes *l* and *m*. We observe that for the mode $l=1$, the error is limited by the tolerance $\epsilon$ of the pressure-correction iterations (see Fig. 3). Hence, we present the convergence of the numerical method for $l=2$ in Fig. 7.

## Conclusions

We have presented a generic C++ expression system for numerically solving partial differential equations (PDEs), particularly as they occur in hydrodynamics. The expression system is based on the parallel computing library OpenFPM [16] for scalable numerical simulations on parallel computing systems.

We demonstrated the use of the presented expression system in three test cases prototypical of biological hydrodynamics simulations. Implementing an incompressible Navier–Stokes solver using a novel mesh-free pressure-correction algorithm required only 156 lines of code. We validated the simulation against data from the literature for the nonlinear lid-driven cavity problem. Changing the numerical method from mesh-free DC-PSE to finite differences on a regular Cartesian grid required changing 21 lines of code. A two-dimensional solver for viscous active polar fluids using a Lagrangian particle method was implemented in 340 lines of code, reusing 50 lines from the previous Navier–Stokes solver. We verified it in a grid convergence study and showed a parallel efficiency of 87% on up to 24 CPU cores. Changing this code to using regular-grid finite differences required changing 20 lines of code. Finally, a simulation of Stokes flow in a three-dimensional ball was implemented in 180 lines of code to demonstrate a simulation in a non-Cartesian domain, validated against an analytical solution. Taken together, these test cases demonstrated significant improvements in developer productivity compared to manually implementing such simulations.

The main limitation of our work is that it can only be used in conjunction with numerical methods that have previously been implemented in the OpenFPM numerics library. At the time of writing, this includes DC-PSE [29], finite differences, the time-integration methods from the Odeint library [25], and the solvers from the PETSc [23] and Eigen [24] libraries. Further numerical methods remain to be implemented or wrapped. Thanks to the separation of concerns achieved by the present framework, however, such implementation can be done independent of the numeric data types of the simulation, the simulation domain dimensionality, or the model equations.

Another drawback is that codes implemented in our expression system require more time to compile than a regular C++ code, because the expressions are parsed and translated at compile time in order to map the computations. The total compile-time overhead depends on the complexity and length of the expressions. For the application examples shown here, compile times roughly doubled when using the expression system. However, this has to be discounted against the time saved when writing and debugging the code.

Further, our expression system does not free the user from carefully choosing the numerical methods appropriate to solve a given PDE and tune their parameters (e.g., time step size, solver tolerance, etc.). It performs no auto-tuning. However, we believe that it provides a good substrate syntax for higher-level languages and problem solving environments [41].

In the future, we plan to extend the presented expression system to include additional numerical methods, such as smoothed particle hydrodynamics [42] and higher-order particle-mesh interpolation [43], and to more general meshes, such as unstructured grids and general triangulated meshes. We also plan to implement numerical methods for the computation of intrinsic derivatives in curved spaces, such as the Laplace-Beltrami operator or the Laplace-de Rham operator [44]. Finally, future work could further improve the user-friendliness and portability of the presented expression system by providing wrappers for scripting languages like Python or by integrating it with domain-specific simulation languages like OpenPME [41].

The presented C++ template expression system for PDEs is available to users as open source, bundled with the test cases presented here, in the OpenFPM library, release 3.2 or newer, available from openfpm.mpi-cbg.de.

## Appendix A: discretization corrected particle strength exchange

Discretization-Corrected Particle Strength Exchange (DC-PSE) is a numerical method for discretizing differential operators on potentially irregularly distributed collocation points [29]. It is a particle method from the numerical class of collocation methods. In contrast to other collocation particle methods, like smoothed particle hydrodynamics (SPH) [42] or Particle Strength Exchange (PSE) [45], however, DC-PSE is fully consistent and achieves the desired order of error convergence for arbitrary linear differential operators discretized at (almost) arbitrary discretization points. DC-PSE effectively generalizes finite difference methods to irregularly distributed as well as moving (i.e., Lagrangian) discretization points, called *particles*, in the sense that the classic finite-difference stencils are recovered analytically in the limit of regular Cartesian particle distributions (see Ref. [29] for proof).

While the details of DC-PSE are well documented elsewhere [29, 36], we here briefly review the main concept for the reader’s convenience. We do so in two dimensions, ${\mathbf {x}}=(x,y)\in {\mathbb {R}}^2$, in order to simplify the notation, noting that the concept also holds in arbitrary-dimensional spaces [29]. In all cases, the purpose of DC-PSE is to discretize a derivative

10 $$\begin{aligned} D^{m,n} = \frac{\partial ^{m+n}}{\partial x^m \partial y^n} , \end{aligned}$$

where *m* and *n* are the order of the derivative in the two spatial dimensions. For example, for $m=1$ and $n=0$, the operator $D^{m,n}$ is the first derivative along *x*, i.e., $\frac{\partial }{\partial x}$.

DC-PSE discretizes the operator $D^{m,n}$ over a set of *N* particles at locations ${\mathbf {x}}_p$, $p=1,\ldots ,N$, by a discrete convolution operator $Q^{m,n}$. The discretization can be derived from a Taylor series expansion requiring that $Q^{m,n} = D^{m,n} + O(h^r)$, where *h* is the average distance between neighboring particles and *r* the desired order of convergence. This results in the discrete operator

11 $$\begin{aligned}&Q^{m, n} f\left( {\mathbf {x}}_p\right) \nonumber \\&\quad =\frac{1}{\epsilon \left( {\mathbf {x}}_p\right) ^{m+n}} \sum _{{\mathbf {x}}_q \in {\mathcal {N}}\left( {\mathbf {x}}_p\right) }\left( f\left( {\mathbf {x}}_q\right) \pm f\left( {\mathbf {x}}_p\right) \right) \eta \left( \frac{{\mathbf {x}}_p-{\mathbf {x}}_q}{\epsilon \left( {\mathbf {x}}_p\right) }\right) \nonumber \\ \end{aligned}$$

when applied to compute the respective derivative of a sufficiently smooth function $f({\mathbf {x}})$ at the location ${\mathbf {x}}_p$ of particle *p*. The convolutional sum on the right-hand side is taken over all particles *q* in the neighborhood ${\mathcal {N}}({\mathbf {x}}_p)$ of particle *p*. The neighborhood is defined by the support of the *operator kernel* $\eta$ and is local with *kernel radius* $\epsilon$. The operator can thus be evaluated in constant time per particle. The sign ± is positive if $(m+n)$ is odd and negative if $(m+n)$ is even.

The operator kernel $\eta$ is determined numerically at runtime such that the required order of convergence is achieved on the specific given particle distribution. From the Taylor expansion used to derive the operator approximation, one can see that the correct order of convergence is achieved if and only if the discrete moments

12 $$\begin{aligned} Z^{i, j}\left( {\mathbf {x}}_{p}\right) =\sum _{{\mathbf {x}}_{q} \in {\mathcal {N}}\left( {\mathbf {x}}_{p}\right) } \frac{\left( x_{p}-x_{q}\right) ^{i}\left( y_{p}-y_{q}\right) ^{j}}{\epsilon \left( {\mathbf {x}}_{p}\right) ^{i+j}}\eta \left( \frac{{\mathbf {x}}_{p}-{\mathbf {x}}_{q}}{\epsilon \left( {\mathbf {x}}_{p}\right) }\right) \nonumber \\ \end{aligned}$$

of the kernel $\eta$ satisfy the conditions

13 $$\begin{aligned} Z^{i, j}\left( {\mathbf {x}}_{p}\right) =\left\{ \begin{array}{ll} i ! j!(-1)^{i+j},&{}\quad i=m, j=n \\ 0,&{}\quad 0<i+j<r+m+n \\ <\infty , &{}\quad \text {otherwise. }\end{array}\right. \end{aligned}$$

For a polynomial kernel, as used in the present work,

14 $$\begin{aligned} \eta ({\mathbf {x}})=\sum _{i, j}^{i+j<r+m+n} a_{i, j} x^{i} y^{j} e^{-x^{2}-y^{2}} \end{aligned}$$

with truncated support $\sqrt{x^{2}+y^{2}}<r_{c}$ (cutoff radius), the coefficients $a_{i,j}$ of the kernel polynomial can then be determined from the moment conditions by solving a small linear system of equations. The kernel radius $\epsilon$ can be chosen freely as required by the spatial resolution of the simulation or to achieve the desired numerical stability.

This linear system is independently solved for each particle *p*, using the appropriate discrete moment from Eq. (13) that accounts for the spatial distribution of the neighboring particles. This way, the discrete moment conditions are always *exactly* fulfilled when evaluated on any particle. This renders DC-PSE fully consistent (i.e., convergent with full order *r* to arbitrarily small errors only limited by the floating-point arithmetics of the computer) on irregular and moving particle distributions. The price one has to pay is the computational overhead of solving for the kernel coefficients $a_{i,j}$ for each particle and every time the particles have moved. Benchmarks have shown, however, that this additional computational cost may be amortized by the gain in accuracy, actually reducing the overall solution time for flow-dominated problems [29]. Due to its inherent adaptivity and the flexibility afforded in placing the collocation points, DC-PSE is also well suited for simulations in non-Cartesian as well as moving or deforming domains.

## Appendix B: incompressible active polar fluids

The passive stress $\sigma ^{(\mathrm {p})}_{\alpha \beta }= \sigma ^{(\mathrm {s})}_{\alpha \beta }+\sigma ^{(\mathrm {e})}_{\alpha \beta }+ \sigma _{\alpha \beta }^{(\mathrm {ant})}$ is decomposed as the sum of the symmetric ($\mathrm {s}$), antisymmetric ($\mathrm {ant}$), and equilibrium ($\mathrm {e}$) stresses. The equilibrium stress, also called the Ericksen stress, is given by

15 $$\begin{aligned} \sigma _{\alpha \beta }^{(\mathrm {e})}=-\frac{\partial f}{\partial \left( \partial _{\beta } p_{\gamma }\right) } \partial _{\alpha } p_{\gamma }, \end{aligned}$$

where *f* is free energy density from Eq. (4). The symmetric stress is the deviatoric part of the symmetric stress tensor (i.e., the total passive symmetric stress). The antisymmetric stress is given by

16 $$\begin{aligned} \sigma _{\alpha \beta }^{(\mathrm {ant})}=\frac{1}{2}\left( p_{\alpha } h_{\beta }-p_{\beta } h_{\alpha }\right) . \end{aligned}$$

Following Eq. (2a), the molecular field $h_\alpha$ is decomposed into the component $h_{\Vert }$ parallel and the component $h_{\perp }$ perpendicular to the local polarity ${\mathbf {p}}$, where $h_{\Vert }$ is derived as a Lagrange multiplier,

17 $$\begin{aligned} h_{\Vert }=-\gamma \left[ \lambda \varDelta \mu -\nu \frac{u_{\mathrm {xx}} p_{\mathrm {x}}^{2}}{p_{\mathrm {x}}^{2}+p_{\mathrm {y}}^{2}}-\nu \frac{u_{\mathrm {yy}} p_{\mathrm {y}}^{2}}{p_{\mathrm {x}}^{2}+p_{\mathrm {y}}^{2}}-2 \nu \frac{u_{\mathrm {xy}} p_{\mathrm {x}} p_{\mathrm {y}}}{p_{\mathrm {x}}^{2}+p_{\mathrm {y}}^{2}}\right] \end{aligned}$$

to enforce $\Vert {\mathbf {p}}\Vert =1$. Equations (2b) and (2d) are then combined to the final component-wise expressions [13]:

18 $$\begin{aligned}&\eta \partial _{x}^{2} v_{\mathrm {x}}+\eta \partial _{y}^{2} v_{\mathrm {x}}-\partial _{x} \Pi +\frac{\nu }{2} \partial _{x}\left[ \frac{\gamma \nu u_\mathrm {x x} p_{\mathrm {x}}^{2}\left( p_{\mathrm {x}}^{2}-p_{\mathrm {y}}^{2}\right) }{p_{\mathrm {x}}^{2}+p_{\mathrm {y}}^{2}}\right] \nonumber \\&\qquad +\frac{\nu }{2} \partial _{x}\left[ \frac{2 \gamma \nu u_{\mathrm {xy}} p_{\mathrm {x}} p_{\mathrm {y}}\left( p_{\mathrm {x}}^{2}-p_{\mathrm {y}}^{2}\right) }{p_{\mathrm {x}}^{2}+p_{\mathrm {y}}^{2}}\right] \nonumber \\&\qquad +\frac{\nu }{2} \partial _{x}\left[ \frac{\gamma \nu u_{\mathrm {yy}} p_{\mathrm {y}}^{2}\left( p_{\mathrm {x}}^{2}-p_{\mathrm {y}}^{2}\right) }{p_{\mathrm {x}}^{2}+p_{\mathrm {y}}^{2}}\right] \nonumber \\&\qquad +\frac{\nu }{2} \partial _{y}\left[ \frac{2 \gamma \nu u_{\mathrm {xx}} p_{\mathrm {x}}^{3} p_{\mathrm {y}}}{p_{\mathrm {x}}^{2}+p_{\mathrm {y}}^{2}}\right] +\frac{\nu }{2} \partial _{y}\left[ \frac{4 \gamma \nu u_{\mathrm {xy}} p_{\mathrm {x}}^{2} p_{\mathrm {y}}^{2}}{p_{\mathrm {x}}^{2}+p_{\mathrm {y}}^{2}}\right] \nonumber \\&\qquad +\frac{\nu }{2} \partial _{y}\left[ \frac{2 \gamma \nu u_{\mathrm {yy}} p_{\mathrm {x}} p_{\mathrm {y}}^{3}}{p_{\mathrm {x}}^{2}+p_{\mathrm {y}}^{2}}\right] \nonumber \\&\quad =-\frac{1}{2} \partial _{y}\left( h_{\perp }\right) +\zeta \partial _{x}\left( \varDelta \mu p_{\mathrm {x}}^{2}\right) +\zeta \partial _{y}\left( \varDelta \mu p_{\mathrm {x}} p_{\mathrm {y}}\right) \nonumber \\&\qquad -\zeta \partial _{x}\left( \varDelta \mu \frac{p_{\mathrm {x}}^{2}+p_{\mathrm {y}}^{2}}{2}\right) - \frac{\nu }{2} \partial _{x}\left( -2 h_{\perp } p_{\mathrm {x}} p_{\mathrm {y}}\right) \nonumber \\&\qquad -\frac{\nu }{2} \partial _{y}\left[ h_{\perp }\left( p_{\mathrm {x}}^{2} -p_{\mathrm {y}}^{2}\right) \right] -\partial _{x} \sigma _{\mathrm {xx}}^{(\mathrm {e})} -\partial _{y} \sigma _{\mathrm {xy}}^{(\mathrm {e})} \nonumber \\&\qquad +\frac{\nu }{2} \partial _{x}\left[ \gamma \lambda \varDelta \mu \left( p_{\mathrm {x}}^{2}-p_{\mathrm {y}}^{2}\right) \right] -\frac{\nu }{2} \partial _{y}\left( -2 \gamma \lambda \varDelta \mu p_{\mathrm {x}} p_{\mathrm {y}}\right) ,\qquad \end{aligned}$$

19 $$\begin{aligned}&\eta \partial _{x}^{2} v_{\mathrm {y}}+\eta \partial _{y}^{2} v_{\mathrm {y}}-\partial _{y} \Pi +\frac{\nu }{2} \partial _{y}\left[ \frac{-\gamma \nu u_{\mathrm {xx}} p_{\mathrm {x}}^{2}\left( p_{\mathrm {x}}^{2}-p_{\mathrm {y}}^{2}\right) }{p_{\mathrm {x}}^{2}+p_{\mathrm {y}}^{2}}\right] \nonumber \\&\qquad + \frac{\nu }{2} \partial _{y}\left[ \frac{-2 \gamma \nu u_{\mathrm {xy}} p_{\mathrm {x}} p_{\mathrm {y}}\left( p_{\mathrm {x}}^{2}-p_{\mathrm {y}}^{2}\right) }{p_{\mathrm {x}}^{2}+p_{\mathrm {y}}^{2}}\right] \nonumber \\&\qquad +\frac{\nu }{2} \partial _{y}\left[ \frac{-\gamma \nu u_{\mathrm {yy}} p_{\mathrm {y}}^{2}\left( p_{\mathrm {x}}^{2}-p_{\mathrm {y}}^{2}\right) }{p_{\mathrm {x}}^{2}+p_{\mathrm {y}}^{2}}\right] \nonumber \\&\qquad +\frac{\nu }{2} \partial _{x}\left[ \frac{2 \gamma \nu u_{\mathrm {x} x} p_{\mathrm {x}}^{3} p_{\mathrm {y}}}{p_{\mathrm {x}}^{2}+p_{\mathrm {y}}^{2}}\right] +\frac{\nu }{2} \partial _{x}\left[ \frac{4 \gamma \nu u_{\mathrm {xx}} p_{\mathrm {x}}^{2} p_{\mathrm {y}}^{2}}{p_{\mathrm {x}}^{2}+p_{\mathrm {y}}^{2}}\right] \nonumber \\&\qquad +\frac{\nu }{2} \partial _{x}\left[ \frac{2 \gamma \nu u_{\mathrm {yy}} p_{\mathrm {x}} p_{\mathrm {y}}^{3}}{p_{\mathrm {x}}^{2}+p_{\mathrm {y}}^{2}}\right] \nonumber \\&\quad =-\frac{1}{2} \partial _{x}\left( -h_{\perp }\right) +\zeta \partial _{y}\left( \varDelta \mu p_{\mathrm {y}}^{2}\right) +\zeta \partial _{x}\left( \varDelta \mu p_{\mathrm {x}} p_{\mathrm {y}}\right) \nonumber \\&\qquad -\zeta \partial _{y}\left( \varDelta \mu \frac{p_{\mathrm {x}}^{2}+p_{\mathrm {y}}^{2}}{2}\right) -\frac{\nu }{2} \partial _{y}\left( 2 h_{\perp } p_{\mathrm {x}} p_{\mathrm {y}}\right) \nonumber \\&\qquad -\frac{\nu }{2} \partial _{x}\left[ h_{\perp }\left( p_{\mathrm {x}}^{2}-p_{\mathrm {y}}^{2}\right) \right] -\partial _{x} \sigma _{\mathrm {yx}}^{(\mathrm {e})}-\partial _{y} \sigma _{\mathrm {yy}}^{(\mathrm {e})}\nonumber \\&\qquad -\frac{\nu }{2} \partial _{y}\left[ \gamma \lambda \varDelta \mu \left( p_{\mathrm {x}}^{2}-p_{\mathrm {y}}^{2}\right) \right] -\frac{\nu }{2} \partial _{x}\left( -2 \gamma \lambda \varDelta \mu p_{\mathrm {x}} p_{\mathrm {y}}\right) . \end{aligned}$$

Together with the incompressibility condition in Eq. (2c), this completes the force balance.
